# Establishment of a multiplex qPCR assay for the detection of pathogens associated with bovine respiratory disease complex

**DOI:** 10.3389/fvets.2025.1594488

**Published:** 2025-04-22

**Authors:** Linghao Li, Qifeng Jiang, Siying Li, Xin Li, Shenghe Sun, Xiyi Wang, Chuangqi Sun, Kun Jia, Shoujun Li

**Affiliations:** ^1^College of Veterinary Medicine, South China Agricultural University, Guangzhou, China; ^2^Guangdong Technological Engineering Research Center for Pet, Guangzhou, China

**Keywords:** bovine respiratory syncytial virus, bovine parainfluenza virus type 3, bovine viral diarrhea virus, bovine adenovirus type 3, *Mycoplasma bovis*, infectious bovine rhinotracheitis virus, multiplex real-time fluorescence quantitative PCR

## Abstract

**Introduction:**

The bovine respiratory disease complex poses a significant threat to the cattle industry, necessitating a multifaceted approach to address its occurrence. The syndrome is caused by various pathogens such as bovine respiratory syncytial virus (BRSV), bovine parainfluenza virus type 3 (BPIV3), bovine viral diarrhea virus (BVDV), bovine adenovirus type 3 (BAV3), *Mycoplasma bovis* (Mb), and infectious bovine rhinotracheitis virus (IBRV). The confluence of these pathogens causes substantial economic losses to the cattle industry. Although preventive and control measures have been implemented, containment of bovine respiratory diseases continues to present a formidable challenge, highlighting the need for innovative diagnostic and intervention strategies.

**Methods:**

In this study, we designed specific primers targeting six conserved pathogen genes (*N* of BRSV, *M* of BPIV3, *5’UTR* of BVDV, *Hexon* of BAV3, *oppF* of Mb, and *gB* of IBRV). Subsequently, we established a multiplexed fluorescent real-time quantitative PCR (qPCR) assay for simultaneous detection of these pathogens.

**Results:**

The developed method exhibited high specificity and sensitivity, with the lowest detection limits for plasmid DNA standards of BRSV, BPIV3, BVDV, BAV3, Mb, and IBRV being 70.1, 40.4, 15.1, 74.4, 69.6, and 4.99 copies/μL, respectively. The coefficients of variation determined by the assay established in this study were <4%, and the amplification efficiency was 93.84%–111.60%, which showed the reliability and stability of the method.

**Discussion:**

The detection rates for BRSV, BPIV3, BVDV, BAV3, Mb, and IBRV were 7.59% (17/224), 11.61% (26/224), 8.04% (18/224), 22.32% (50/224), 27.23% (61/224), and 8.04% (18/224), respectively. All 224 cows were cases of natural disease. Fifty-six diseased cattle were infected with a mixture of two or more of the six pathogens at a mixed infection rate of 25% (56/224). Therefore, this study successfully developed a highly efficient, rapid, specific, and sensitive multiplex qPCR method to detect major pathogens associated with bovine respiratory diseases. This advancement is expected to significantly influence the future of the cattle industry and serve as a valuable reference for subsequent research in this field.

## 1 Introduction

The bovine respiratory disease complex (BRDC) is an umbrella term encompassing a spectrum of respiratory ailments in cattle arising from the interplay between various pathogens and environmental factors. The main etiological agents implicated in BRDC include viruses, bacteria, and mycoplasmas (1, 2). In this study, we focused on six key pathogens, namely bovine respiratory syncytial virus (BRSV), bovine parainfluenza virus type 3 (BPIV3), bovine viral diarrhea virus (BVDV), bovine adenovirus type 3 (BAV3), *Mycoplasma bovis* (Mb), and infectious bovine rhinotracheitis virus (IBRV).

BRSV, a member of the family *Paramyxoviridae,* subfamily *Pneumovirinae,* and genus *Pneumovirus* (3), is a single-stranded negative-sense RNA virus. It possesses an enveloped capsid and an unsegmented genome of length approximately 15 kilobases (kb). BRSV was initially isolated from affected cattle in 1970 (4), and in recent years isolations of the pathogen have been reported from China and Turkey (5, 6). Turkish researchers revealed the relationship between calf age, season, and management conditions and BRSV infection rates, and found variations in the G protein of the virulent strains through gene sequence analysis, which may be associated with antigenic changes and provide a basis for regional prevention and control (7). BPIV3 belongs to the family *Paramyxoviridae,* subfamily *Paramyxovirinae,* and genus *Respirovirus,* and is an unsegmented, single-stranded, negative-sense RNA virus, The viral genome is approximately 15 kb in length. BPIV3 often exacerbates the clinical condition of cattle by co-circulating with other pathogens (8, 37). BVDV, classified within the family *Flaviviridae* and genus *Pestivirus*, is a single-stranded, positive-sense RNA virus that possesses an enveloped capsid. BVDV is genetically diverse, with three recognized genotypes: BVDV-1 (Pestivirus A), BVDV-2 (Pestivirus B), and HoBi-like virus (Pestivirus H), each harboring a genome of approximately 12.3 kb (10). BVDV can cause immunosuppression in cattle, predisposing them to secondary infections by other pathogens. In utero infections are particularly concerning, as they can lead to vertical transmission of the pathogen, perpetuating the cycle of infection within the herd (11). Epidemiological studies have shown that seropositivity in animal populations tends to increase with age due to long-term colonization of the virus in the host, suggesting a significant positive correlation between the cycle of infection and the duration of exposure of individuals (12). Abnormal interleukin levels and reduced antioxidant enzyme activity in the serum of cattle naturally infected with BVDV suggest a synergistic role of the inflammatory cascade response and oxidative damage in disease progression (13). BAV3, a member of the family *Adenoviridae* and genus *Mastadenovirus,* is a non-encapsulated, linear, double-stranded DNA virus (14). It is recognized as a significant pathogen in bovine respiratory diseases, with a genome length of 34.4 kb (9). Mb, a prokaryotic pathogen, is the main etiological agent of epidemics characterized by bronchopneumonia, mastitis, and arthritis, and is a major cause of bovine respiratory disease (15, 16). Mb disrupts the mammary epithelial cell barrier and induces a persistent inflammatory response through virulence factors such as adhesion proteins (17), and its genetic diversity (18) may influence differences in pathogenicity of regionally endemic strains. IBRV, a member of the family *Herpesviridae,* subfamily *Alphaherpesvirinae,* and genus Var*icellovirus*, is an enveloped, double-stranded DNA virus with a genome length of approximately 138 kb (19).

Because the clinical symptoms of these pathogens are highly similar, traditional single detection methods such as common PCR and pathogen isolation have limitations such as low efficiency, high cost, and susceptibility to leakage, which make it difficult to meet the needs of rapid clinical diagnosis and precise prevention and control. In recent years, the development of efficient multiplex qPCR detection system has become a research hotspot for the characteristics of BRDC pathogen mixed infection. Rapid advances in molecular biology techniques have provided new tools for pathogen detection and virulence factor analysis. For example, qPCR and loop-mediated isothermal amplification (LAMP)-based methods have significantly improved the sensitivity and specificity of *Mycoplasma bovis* detection (20). Serum biomarkers such as H-FABP and NT-proBNP have been shown to be effective in assessing myocardial injury associated with bovine respiratory disease, providing new ideas for early clinical diagnosis (21). Rapid test reagents for BVDV-specific antibodies (22) enable immediate screening in the field and shorten the diagnostic window period. To cope with the diversity of BRDC pathogens, multiplex real-time PCR technology significantly improves detection efficiency by simultaneously detecting multiple pathogens in a single reaction. Studies have shown that regular monitoring of nasal swab samples from healthy calves can detect low load pathogens before the onset of clinical signs, providing a window period for intervention (23). A 2019–2023 epidemiological survey in Canada revealed that the most common viruses locally were bovine coronavirus and bovine respiratory syncytial virus, and the most common bacteria were *Pasteurella multocida* and *Mannheimia haemolytica*, as revealed by multiplex qPCR analysis (24), which provides data to support vaccine development and control strategy development. Zhang et al. developed a one-step quintuplex qPCR assay capable of detecting five important bovine respiratory pathogens (25). The qPCR technique performed better in terms of both sensitivity and specificity when compared with traditional culture or immunological methods.

In this study, we developed a qPCR assay using specific primers and probes designed for the conserved sequences of six BRDC-associated pathogens. We optimized the reaction system and amplification conditions by grouping three RNA viruses into reaction system 1 for amplification, and two DNA viruses and *Mycoplasma bovis* into reaction system 2 for amplification. This method allows for the simultaneous detection of these six main pathogens, thus simplifying the diagnostic process. Herein, we amplified six pathogens using a dual-reaction system and classified them according to the type of extracted nucleic acid (DNA or RNA). Dividing the six pathogens into two groups reduces the complexity of the reaction system and minimizes competition between primers. Most qPCR instruments support limited fluorescence channels, dual system can be compatible with more conventional instruments, lowering the hardware threshold. The dual-system qPCR achieves a balance between sensitivity, specificity, and equipment compatibility, which is especially suitable for BRDC, a scenario where multiple pathogens need to be detected simultaneously and resources are limited.

## 2 Materials and methods

### 2.1 Primer and probe design

The *N* protein of BRSV demonstrates high evolutionary conservation among viral proteins (26). Comparative phylogenetic analysis reveals that the *M* gene of BPIV3 exhibits relatively lower resolution compared to its *HN*, *P*, and *N* genes, which may be attributed to the *M* gene’s significant sequence conservation (27). In contrast, the *5’-UTR* of BVDV has been established as the preferred target for pathogen identification due to its genetic stability (28). Furthermore, molecular characterization of BAV3 identifies the *hexon* gene as encoding both the major antigenic determinant and one of the most conserved genomic regions (29). The *oppF* gene is one of the key component genes of the oligopeptide permease system and is responsible for encoding ATP-binding proteins. This basal metabolic function makes the gene highly conserved in evolution with a low mutation rate, making it suitable as a stable target for detection. Bashiruddin et al. established a PCR method for the ATP-binding protein *oppD/F* gene as a target gene (30). The *gB*, the main immunoantigen in the viral envelope, is a highly conserved protein and is commonly used as a pathogen diagnostic for IBRV (31).

A comparative analysis was conducted on 12 virulent strain sequences of BRSV, 12 of BPIV3, 20 of BVDV, 6 of BAV3, 15 of Mb, and 10 of IBRV, as recorded in the National Center for Biotechnology Information (NCBI) database. Therefore, specific primers and probes were designed targeting the *N* gene of BRSV, *M* gene of BPIV3, *5’UTR* gene of BVDV, *Hexon* gene of BAV3, *oppF* gene of Mb, and *gB* gene of IBRV (GenBank accession number: MT861050.1, OR597585.1, MN417910.1, JN381195.1, AF130119.1, and JX898220.1, respectively) (Table 1), and analyzed by Primer-BLAST (NCBI) for their specificity. Positions with fewer mutations in multiple sequences were selected for specific primer and probe design using Primer Premier 5 and Oligo 7. All primers and probes were synthesized by Shanghai Sangon Biological (Shanghai, China).

**Table 1:** Primer and probe information and qPCR reaction systems.

Reaction system (20 μL)	Target	Gene	Sequence (5′–3′)	Size (bp)	reaction volume (10 μmol/L)	Other components
1	BRSV	*N*	TTGTCACCTGTACTACGTTGAAT	93	0.7 μL (0.35 μM)	2 × Probe Master mix: 10 μL; cDNA template:2 μL; ddH_2_O:3.3 μL
			GGCTCTTAGCAAGGTCAAACTA		0.7 μL (0.35 μM)	
			FAM-CTGGTTGACAACAGTTGRTCCTTGTTG-MGB		0.1 μL (0.05 μM)	
	BPIV3	*M*	AATAGGTGACCCACCCAAAC	106	0.8 μL (0.4 μM)	
			CAGATCACTGACACTCCCATATC		0.8 μL (0.4 μM)	
			VIC-TTTGAGATGGAGCGGTCAAAGGACA-MGB		0.1 μL (0.05 μM)	
	BVDV	*5’UTR*	GTCTAACCGACTGCTACGAATAC	87	0.7 μL (0.35 μM)	
			GTGCCATGTACAGCAGAGAT		0.7 μL (0.35 μM)	
			CY5-TTTAGTAGCAATACAGTGGGCCTCTGC-MGB		0.1 μL (0.05 μM)	
2	BAV3	*Hexon*	ACCTACGAGTGGTCCTTTAGA	111	0.8 μL (0.4 μM)	2 × Probe Master mix: 10 μL; cDNA template: 2 μL; ddH_2_O: 3.1 μL
			GTAGAGGTTGACGCTGGTAAT		0.8 μL (0.4 μM)	
			FAM-AGTGCTCTGAAGGATCATGTTTACGTCC-MGB		0.1 μL (0.05 μM)	
	Mb	*oppF*	GCTGTTGATGGTGTGTCATTT	93	0.7 μL (0.35 μM)	
			TGAACGTCCAACAGTGGTT		0.7 μL (0.35 μM)	
			VIC-CGGTCTAATTGGTGAGTCAGGAAGTGG-MGB		0.1 μL (0.05 μM)	
	IBRV	*gB*	TGCTCGACTACAGCGAGATA	92	0.8 μL (0.4 μM)	
			CATATTGCCGTCCGTCTTGA		0.8 μL (0.4 μM)	
			CY5-CACGAGCTCCGGTTCTACGACATTG-MGB		0.1 μL (0.05 μM)	

### 2.2 Sample preparation

The collected nasal swabs were thoroughly mixed with 1 mL of phosphate-buffered saline solution and were centrifuged by centrifuge at 13,523 g (Eppendorf Ltd., Hamburg, Germany) for 1 min at 4°C. Subsequently, nucleic acids were extracted using a Virus DNA/RNA Extraction Kit 2.0 (Nanjing Vazyme Biotech Co., Ltd., Nanjing, China) with a fully automated nucleic acid extractor (Nanjing Vazyme Biotech Co., Ltd., Nanjing, China), following the RM-401 default program. RNA extracted from nasal swabs was reverse-transcribed using HiScript III All-in-one RT SuperMix (Nanjing Vazyme Biotech Co., Ltd., Nanjing, China). DNA and cDNA were stored at −20°C for further analysis.

### 2.3 Preparation of standard plasmids

The amplification products of the *N* gene (2–325, 324 bp) of BRSV, *M* gene (1–335, 335 bp) of BPIV3, *5’UTR* gene (39–280, 242 bp) of BVDV, *Hexon* gene (1,414–1,748, 335 bp) of BAV3, *oppF* (1,913–2,562, 650 bp) gene of Mb, and *gB* gene (1–650, 650 bp) of IBRV were cloned into the pMD18-T vector [Takara Biotechnology (Dalian) Co., Ltd., Dalian, China]. The recombinant plasmids were then transformed into DH5α competent cells (Guangzhou Xinkailai Biotechnology Co., Ltd., Guangzhou, China). Single-positive colonies were selected under sterile conditions, inoculated into ampicillin-resistant medium, and cultured, and their bacterial lysates were submitted to Shanghai Sangon Biotechnology for sequencing. The correctly sequenced positive plasmids were designated as pMD18-T-BRSV, pMD18-T-BPIV3, pMD18-T-BVDV, pMD18-T-BAV3, pMD18-T-Mb, and pMD18-T-IBRV. Plasmid DNA was extracted from the transformed cells using the EndoFree Mini Plasmid Kit II (Tiangen Biochemical Technology, Beijing, China) and stored at −20°C for subsequent experiments.

### 2.4 Reaction system procedure and optimization

First, we mixed six plasmids of 10^6^ copies/μL in an ep tube and mixed them as our templates. The different primers and probes were aligned and combined for amplification with the following procedure: initial denaturation at 95°C for 5 min, followed by 45 cycles of denaturation at 95°C for 10 s and annealing/extension at 60°C for 30 s, with fluorescence signal acquisition at 60°C. Determine whether cross-reactivity occurs between different primers and probes.

In this study, we established two PCR reaction systems for the detection of six pathogens. Pathogens with RNA nucleic acids, namely BRSV, BPIV3, and BVDV, were used in PCR reaction system 1. Conversely, pathogens with DNA nucleic acids, including BAV3, Mb, and IBRV, were used in the PCR reaction system 2. The same protocol was followed for both reaction systems.

The reaction systems, with a total volume of 20 μL, comprised six pairs of primers, six probes, sterile double-distilled water, 2 μL of DNA/cDNA template, and 10 μL of the AceQ qPCR Probe Master mix (Nanjing Vazyme Biotech Co., Ltd., Nanjing, China). Amplification and analysis were performed using LightCycler 480 II real-time fluorescent qPCR (Roche Diagnostics (Shanghai) Limited, Shanghai, China).

We used 10^6^ copies/μL of standard plasmids for BRSV, BPIV3, BVDV, BAV3, Mb, and IBRV as templates. The concentrations of primers and probes were optimized using the following thermal cycling conditions: initial denaturation at 95°C for 5 min, followed by 45 cycles of denaturation at 95°C for 10 s and annealing/extension at 60°C for 30 s, with fluorescence signal acquisition at 60°C. After determining the optimal concentrations of primers and probes, we evaluated seven annealing temperature gradients ranging from 56 to 62°C to calibrate the annealing temperature.

### 2.5 Establishing standard curves

A 10-fold serial dilution of the standard plasmid was performed, ranging from 10^8^ to 10^0^ copies/μL. Real-time fluorescent quantitative PCR amplification of the diluted standard plasmid DNAs was performed in triplicate for each dilution using the optimized reaction system and protocol. The logarithm of the initial copy number of the standard plasmid DNA is plotted on the X-axis, whereas the cycle threshold (Ct) values obtained from real-time fluorescent quantitative PCR are plotted on the Y-axis. Standard curves were generated using GraphPad Prism 8 (GraphPad Software, California, United States).

### 2.6 Sensitivity test

A 10-fold serial dilution of the standard plasmid, ranging from 10^5^ to 10^0^ copies/μL, was performed. Using an optimized reaction system and protocol, real-time fluorescent qPCR amplification was performed on diluted standard plasmid samples, with sterile double-distilled water serving as a negative control. This process allowed us to determine the minimum detectable copy number for each pathogen. Graphical representations of the data were created using GraphPad Prism 8 (GraphPad Software, California, United States). The reaction was carried out with 2 μL of upstream and downstream primers (no probe required), 10 μL of 2 × Taq PCR Star Mix (Kangrun Chengye Biotechnology (Beijing) Limited, Beijing, China) and 2 μL of plasmid as templates, supplemented with 4 μL of water to a 20 μL system and performed with the following program: 5 min at 95°C; 35 cycles of 30 s at 95°C, 30 s at 56°C, 15 s at 72°C; and 5 min at 72°C. Finally, a comparison of the sensitivity of qPCR and PCR was made.

### 2.7 Specificity test

In this study, we assessed the specificity of a multiplex real-time fluorescent quantitative PCR assay using optimized reaction conditions and systems with DNA/cDNA templates from BRSV, BPIV3, BVDV, BAV3, Mb, IBRV, bovine torovirus (BToV), bovine enterovirus (BEV), bovine norovirus (BNoV), bovine rotavirus (BRV), and bovine ephemeral fever virus. Sterile double-distilled water and laboratory-preserved samples of healthy cattle was used as the negative control. All templates were verified by PCR or viral isolation methods to confirm their identity. The resulting data were graphically represented and analyzed using GraphPad Prism 8 (GraphPad Software, California, United States).

### 2.8 Repeatability test

To assess the reproducibility of the assay developed in this study, we selected 10^7^, 10^6^, and 10^5^ copies/μL, and the plasmid copy number corresponding to a Ct value of around 30 and the lowest limiting copy number for the assay. Three replicates of both intra-and inter-assay experiments were conducted using these diluted plasmids with the optimized reaction system and protocol. The average Ct values, standard deviations (SD), and coefficients of variation (CV) were calculated. The reproducibility and stability of the assay were subsequently evaluated based on the obtained CV values.

### 2.9 Sample testing

In this study, a total of 224 nasal swabs were collected from cattle with BRDC in Guangdong Province, China. The samples and healthy bovine nasal swabs and serum samples were subjected to nucleic acid extraction and reverse transcription according to the method described in Section 2.2, and then the extracted nucleic acids were used as templates for amplification according to the optimized reaction system and procedure.

### 2.10 Comparative analysis of detection performance

We selected some positive samples tested and randomly selected samples that tested negative in this study for comparison with a standardized qPCR test kit (Shenzhen Anieasy Biotechnology Co., Ltd., Shenzhen, China) for bovine respiratory disease complex as well as a patented primer and probe for BAV3 (patent no. CN200910062095.9).

## 3 Results

### 3.1 Preparation of standard plasmids

Standard plasmids were extracted and their corresponding concentrations were determined using a NanoDrop One ultramicrospectrophotometer (Thermo Fisher Scientific, Shanghai, China). The measured concentrations of BRSV, BPIV3, BVDV, BAV3, Mb and IBRV were 231, 133.5, 483, 246, 254 and 182 ng/μL. The copy numbers were calculated using the following formula:


$$\begin{array}{l} \mathrm{copy}\;\mathrm{number}\left(\mathrm{copies}/\mathrm{\mu L}\right)=\\ \frac{\mathrm{Concentration}\left(\mathrm{ng}/\mathrm{\mu L}\right)\times 10^{-9}\times 6.022\times 10^{23}}{\mathrm{DNAlength}\times 660} \end{array}$$

The copy numbers of standard plasmids for BRSV, BPIV3, BVDV, BAV3, Mb, and IBRV were calculated to be 7.01 × 10^10^, 4.04 × 10^10^, 1.51 × 10^11^, 7.44 × 10^10^, 6.96 × 10^10^, and 4.99 × 10^10^ copies/μL, respectively. Furthermore, the PCR amplification and sequencing results proved that the positive standard plasmid construction was successful (Supplementary Figures 1, 2).

### 3.2 Reaction system procedure and optimization

It has been verified that no cross-reactivity occurs between mismatched primers and probes, they exhibit no amplification (Table 2). In this study, we applied the same reaction procedure and divided it into two reaction systems for real-time fluorescence qPCR amplification according to the different extracted nucleic acids. The optimized reaction systems are listed in Table 1. The optimal annealing temperature for both reaction systems was 61°C.

**Table 2:** Primer-probe cross-reaction results.

Probe components	Primer components					
	BRSV-Primer	BPIV3-Primer	BVDV-Primer	BAV3-Primer	Mb-Primer	IBRV-Primer
BRSV-Probe	+	−	−	−	−	−
BPIV3-Probe	−	+	−	−	−	−
BVDV-Probe	−	−	+	−	−	−
BAV3-Probe	−	−	−	+	−	−
Mb-Probe	−	−	−	−	+	−
IBRV-Probe	−	−	−	−	−	+

### 3.3 Standard curve and sensitivity analysis

**Figure 1:**
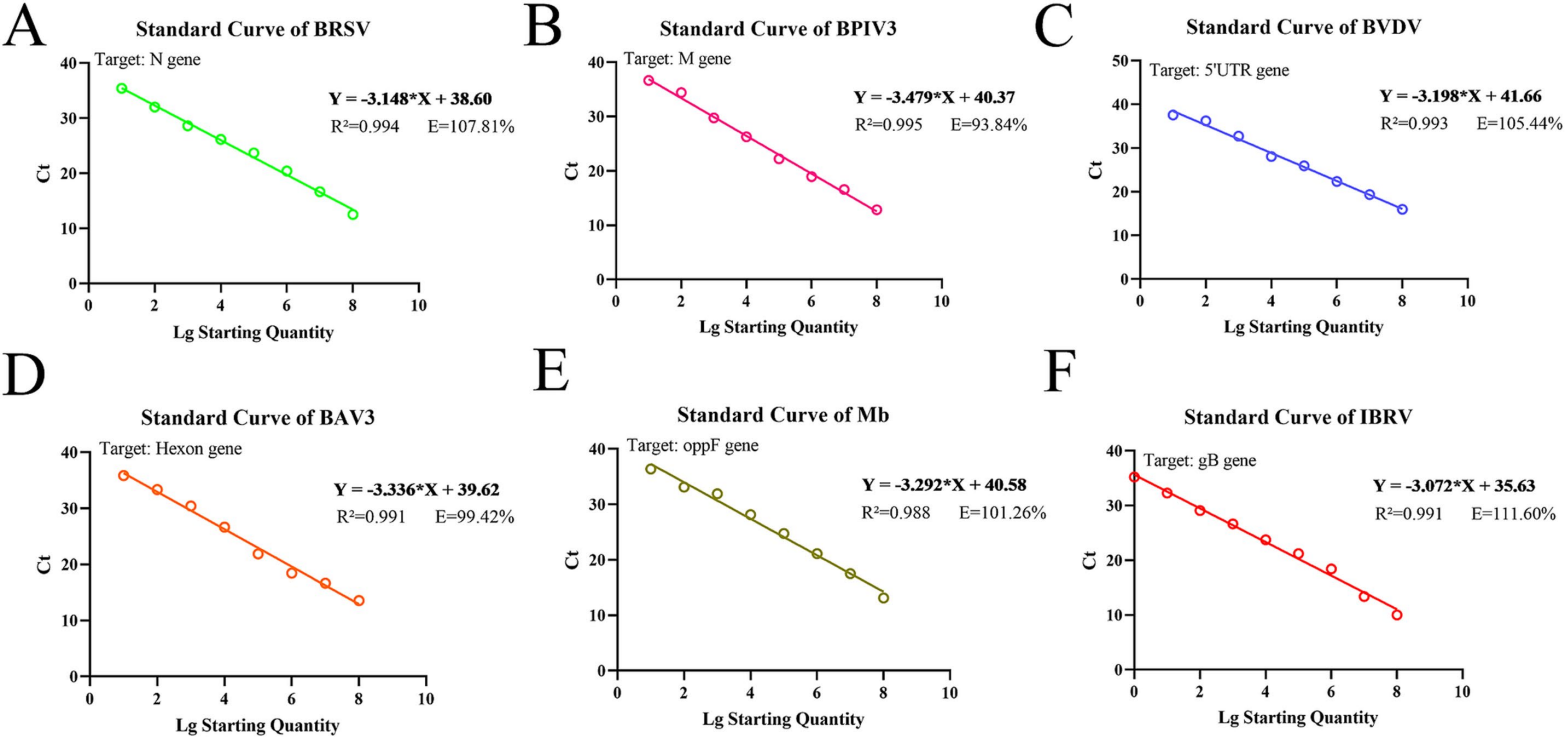
Standard curves based on the qPCR amplification of six pathogenic plasmids. **(A)** BRSV; **(B)** BPIV3; **(C)** BVDV; **(D)** BAV3; **(E)** Mb; **(F)** IBRV.

Standard curves were established based on the amplification results of real-time fluorescence qPCR on positive standard plasmids, ranging from 10^8^ copies/μL to 10^0^ copies/μL (Figure 1; Supplementary Figure 3). The method showed a good amplification efficiency, with a good linear relationship between log starting quantity (X-axis) and Ct (Y-axis), as follows: BRSV expression, Y = −3.148X + 38.60, E = 107.81%, R^2^ = 0.994; BPIV3 expression, Y = −3.479X + 40.37, E = 93.84%, R^2^ = 0.995; BVDV expression, Y = −3.198X + 41.66, E = 105.44%, R^2^ = 0.993; BAV3 expression, Y = −3.336X + 39.62, E = 99.42%, R^2^ = 0.991; Mb expression, Y = −3.292X + 40.58, E = 101.26%, R^2^ = 0.988; and IBRV expression, Y = −3.072X + 35.63, E = 111.60%, R^2^ = 0.991.

The standard positive plasmids of the six pathogens were used as templates for sensitivity tests of the assay established in this study (Supplementary Figure 4). The lowest detectable levels of BRSV, BPIV3, BVDV, BAV3, Mb, and IBRV were 70.1, 40.4, 15.1, 74.4, 69.6, and 4.99 copies/μL, respectively. In contrast, none of the negative controls showed amplification. The detection limits of traditional PCR for BRSV, BPIV3, BVDV, BAV3, Mb, and IBRV were 7.01 × 10^3^, 4.04 × 10^4^, 1.51 × 10^5^, 7.44 × 10^3^, 6.96 × 10^5^, and 4.99 × 10^4^ copies/μL, respectively (Table 3; Supplementary Figure 5). Compared to traditional PCR, the sensitivity of our detection method increased 100–10,000 times.

**Table 3:** Comparison of the sensitivity of qPCR and PCR to positive standard plasmids.

Plasmid concentration (copies/μL)	BRSV		BPIV3		BVDV		BAV3		Mb		IBRV	
	PCR	qPCR	PCR	qPCR	PCR	qPCR	PCR	qPCR	PCR	qPCR	PCR	qPCR
10^8^	+	+	+	+	+	+	+	+	+	+	+	+
10^7^	+	+	+	+	+	+	+	+	+	+	+	+
10^6^	+	+	+	+	+	+	+	+	+	+	+	+
10^5^	+	+	+	+	+	+	+	+	+	+	+	+
10^4^	+	+	+	+	−	+	+	+	−	+	+	+
10^3^	+	+	−	+	−	+	+	+	−	+	−	+
10^2^	−	+	−	+	−	+	−	+	−	+	−	+
10^1^	−	+	−	+	−	+	−	+	−	+	−	+
10^0^	−	−	−	−	−	−	−	−	−	−	−	+

### 3.4 Specificity analysis

**Figure 2:**
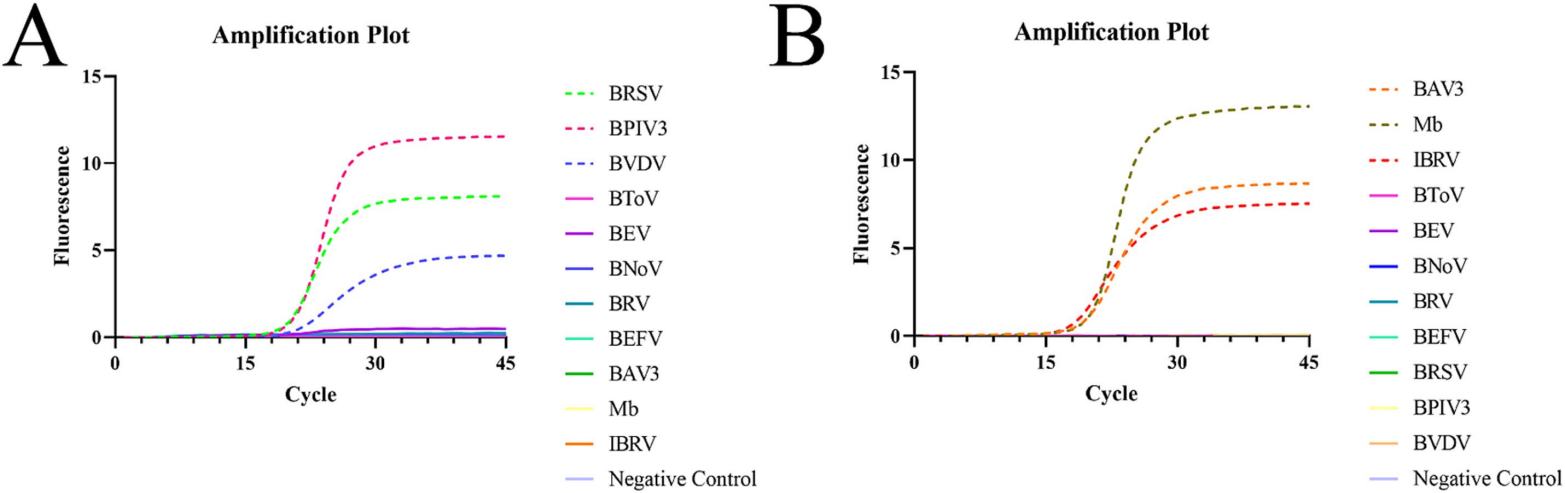
Specific amplification plots based on qPCR amplification of the six pathogenic plasmids. **(A)** Reaction system 1. **(B)** Reaction system 2.

Real-time fluorescence qPCR amplification was performed by adding DNA/cDNA from different pathogens to the corresponding reaction system using the same reaction program (Figure 2). The results showed that in reaction system 1, the instrument only detected the amplification and fluorescence signals of BRSV, BPIV3, and BVDV and did not detect the non-specific amplification of other pathogens; in reaction system 2, the instrument only detected the amplification and fluorescence signals of BAV3, Mb, and IBRV, and did not detect the non-specific amplification of other pathogens. No fluorescence signal or amplification was observed in the negative control wells. This indicates that the method has good specificity.

Next, three intra-and inter-assays were performed using different concentrations of the six plasmids (Table 4). Overall, the range of CVs for BRSV, BPIV3, BVDV, BAV3, Mb, and IBRV was 0.38–2.44, 0.15–3.87, 1.22–3.08, 0.05–2.25, 0.33–2.13, and 0.67–3.24%, respectively. All CVs were <4%, indicating that this multiplex real-time fluorescence qPCR method has good repeatability and stability. The CV was calculated using the following formula:


$$\mathrm{coefficient}\;\mathrm{variation}=\frac{\mathrm{standard}\;\mathrm{deviation}\left(SD\right)}{\mathrm{Mean}\left(MN\right)}\times 100\%$$

**Table 4:** Reproducibility of the multiplex real-time fluorescence quantitative PCR method established in this study.

Target	Plasmid concentration (copies/μL)	Intra-assay		Inter-assay			
		MN	SD	CV (%)	MN	SD	CV (%)
BRSV	10^7^	16.58	0.11	0.66	16.77	0.41	2.44
	10^6^	20.52	0.09	0.44	20.49	0.15	0.73
	10^5^	23.67	0.09	0.38	23.14	0.42	1.82
	10^2^	31.92	0.08	0.25	31.73	0.33	1.04
	10^1^	35.80	0.56	1.56	35.89	0.46	1.28
BPIV3	10^7^	16.14	0.19	1.18	16.79	0.65	3.87
	10^6^	19.68	0.03	0.15	19.45	0.22	1.13
	10^5^	22.85	0.06	0.26	23.05	0.26	1.13
	10^3^	30.20	0.11	0.36	30.51	0.55	1.80
	10^1^	36.36	0.38	1.05	36.68	0.67	1.83
BVDV	10^7^	18.49	0.29	1.57	18.50	0.57	3.08
	10^6^	22.08	0.27	1.22	22.08	0.36	1.63
	10^5^	25.31	0.31	1.22	25.56	0.52	2.03
	10^3^	31.45	0.64	2.03	31.77	0.71	2.23
	10^1^	37.47	0.82	2.19	37.84	0.67	1.77
BAV3	10^7^	16.03	0.36	2.25	15.89	0.22	1.38
	10^6^	19.86	0.01	0.05	20.06	0.16	0.80
	10^5^	22.75	0.05	0.22	23.05	0.23	1.00
	10^2^	32.84	0.12	0.37	32.70	0.56	1.71
	10^1^	35.32	0.20	0.57	35.94	0.81	2.25
Mb	10^7^	16.50	0.29	1.76	16.41	0.35	2.13
	10^6^	20.32	0.14	0.69	20.58	0.12	0.58
	10^5^	24.30	0.15	0.62	24.50	0.08	0.33
	10^3^	30.35	0.26	0.86	30.21	0.32	1.06
	10^1^	36.57	0.25	0.68	36.86	0.32	0.87
IBRV	10^7^	12.86	0.14	1.09	13.28	0.43	3.24
	10^6^	17.94	0.17	0.95	18.06	0.28	1.55
	10^5^	22.43	0.15	0.67	22.66	0.22	0.97
	10^1^	31.52	0.38	1.21	31.76	0.39	1.23
	10^0^	36.15	0.41	1.13	36.48	0.38	1.04

### 3.5 Bovine nasal swab and serum sample testing

**Figure 3:**
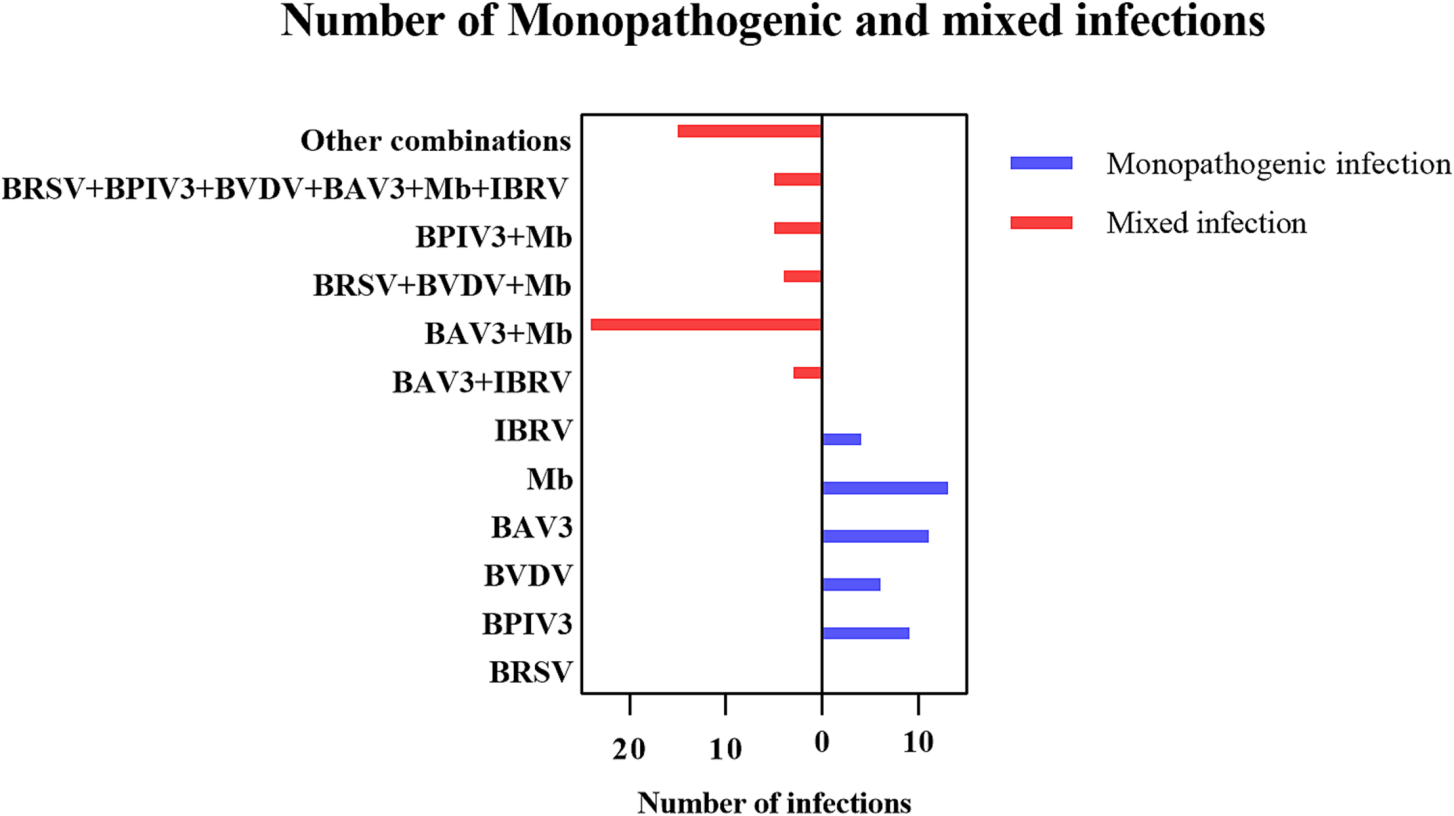
Number of single and mixed infections.

In this study, 224 samples from BRDC-affected cattle were tested, resulting in 99 positive samples, including 17 BRSV, 26 BPIV3, 18 BVDV, 50 BAV3, 61 Mb, and 18 IBRV samples. The detection rates were 7.59% (17/224), 11.61% (26/224), 8.04% (18/224), 22.32% (50/224), 27.23% (61/224), and 8.04% (18/224) respectively. The results showed that 56 cattle had mixed infections to varying degrees, with a mixed infection rate of 25% (Figure 3).

### 3.6 Comparative analysis of detection performance

The assay was performed with the same qPCR instrument and samples, using the method established in this study, standard kits, and proprietary primers and probes for BAV3. The results showed that the assay established in this study was consistent with the positive detection rate of the kit and the patented primer and probe, and the compliance rate was 100%.

## 4 Discussion

The etiological diversity and complexity of BRDC impose a substantial economic burden on the cattle industry. This burden manifests itself primarily through decreased productivity of the affected cattle, significant economic losses attributable to mortality, and escalation of risks associated with farming practices (1, 32). Such surveillance is crucial for ensuring the long-term stable economic development of cattle farms. Therefore, the objective of this study was to develop a dual-reaction system qPCR assay that is efficient, sensitive, and robust for the detection of these key pathogens, thus augmenting preventive and control measures for bovine respiratory diseases.

The templates used in the assay developed in this study were all plasmids, and the target sequences inserted in the plasmids were identical to the viral genome sequences in the publicly available database. It has been shown that plasmid standards can be used to establish initial detection limits and linear ranges for qPCR methods. The non-use of real viral samples is a limitation of this study. Therefore, we will supplement with experiments on the detection ability of the assay when virus samples are obtained.

Recently, various assays have been used to detect pathogens associated with BRDC (25, 33–35). However, the present study introduces an innovative multiplexed qPCR assay that can be categorized into two distinct systems based on the type of nucleic acids extracted from pathogens. Specifically, the assay targets three RNA extracted from pathogens (BRSV, BPIV3, and BVDV) in reaction system 1 and three DNA extracted from pathogens (BAV3, Mb, and IBRV) in reaction system 2. This dual-system approach allows simultaneous amplification under identical reaction conditions. The assay established by Kishimoto et al. (33) was able to detect 17 pathogens with very high throughput, but lower amplification efficiencies (< 90%) for some pathogens may have an impact on the results, and the complexity of the primers and probes raises the likelihood of cross-reactivity, with higher demands on the design of primers and probes. Moreover, their assay may be adapted to higher-end equipment. The one-step qPCR assay developed by Zhang et al. (25) efficiently detects both DNA pathogens and RNA pathogens in a single tube. In order to avoid the potential interference of reverse transcriptase with DNA detection, we separated the RNA pathogen and DNA pathogen into two systems, which improved the stability of the assay and enabled the simultaneous detection of six pathogens. The limitation of this study in comparison to them is also that the separate reverse transcription step may increase the time of sample nucleic acid acquisition. Splitting the amplification into two tubes reduces the difficulty of analysis, but slightly increases the number of steps.

Since the qPCR instrument can acquire four fluorescent signals and the instrument fluorescence channels are limited, we chose to use two reaction systems to realize the detection of the six pathogens. The disadvantages are that the dual reaction system requires double reagents, consumables and instrument occupancy time, which leads to an increase in the cost of the experiment, and the need to optimize and validate the experimental conditions of the two systems separately, which increases the risk of human error. The advantage of the dual-reaction system is that it covers a wider spectrum of pathogens through the combined application, while optimizing the reaction conditions to solve the potential problem of primer cross-interference in multiplexed assays. Splitting the six targets into two sets of assays reduces the risk of cross-reactivity between primer and probe components. Using a dual-reaction system qPCR approach, this technique reduces the detection time by at least half an hour compared to conventional methods. This avoids the inefficiencies associated with single pathogen detection, thus improving the efficiency and speed of disease diagnosis.

The proportions of samples positive for BRSV, BPIV3, BVDV, BAV3, Mb, and IBRV detected in this study were 7.59, 11.61, 8.04, 22.32, 27.23, and 8.04%, respectively. The detection of Mb in BRDC cases suggests that it may be widely colonized in cattle through continuous detoxification and vertical transmission. The high prevalence of BAV3 may be related to immunosuppression due to calf weaning stress, and its latent infection properties may exacerbate synergistic pathogenicity with other pathogens. The prevalence of other pathogens suggests that they remain important causative agents of BRDC. In particular, persistently infected individuals with BVDV may be a hidden source of transmission, and screening needs to be strengthened by a strategy that combines antigen detection with antibody surveillance. Moreover, among these positive samples, 56 cases of mixed infections were observed, which might be related to the increased risk of pathogen exposure under high-density feeding pattern, involving different permutations of pathogens, which implies the complexity of BRDC and the diversity of the associated pathogens, and confirms the difficulty of prevention and control of BRDC. In terms of pathogen combinations, Mb and BAV3 had the highest percentage of co-infection, accounting for 42.9% (24/56) of the mixed infections, suggesting a significant propensity for synergistic infection. Extreme mixed infections in which all six pathogens were infected (5/56) occurred in pastures with incomplete immunization and intensive cattle farming, suggesting that immunosuppression by pathogens such as BVDV and BAV3 is a central factor in multiple infections. The total detection rate of these pathogens is low, suggesting that some diseased cattle may have been infected by bacteria such as *Pasteurella multocida* and *Mannheimia haemolytica* (36). In response to these cases, medication and control of environmental humidity can reduce *Mycoplasma* infections as well as aerosol transmission of other pathogens. Severe cases can be urgently removed by quarantine and herd immunization of susceptible cattle in the herd.

In this study, based on qPCR detection of samples from Guangdong Province, the mixed infection pattern of BRDC was preliminarily revealed, but there were regional limitations. The hot and humid climate of Guangdong may contribute to the enhancement of BAV3 and Mb adaptation, and there may be differences in the pathogen combinations in other dry regions; Guangdong has a high proportion of intensive farming, and in the future, we can also compare the characteristics of mixed infections in provinces with predominantly free-range pastures, such as Inner Mongolia. This method is suitable for complex cases that require simultaneous screening for mixed *Mycoplasma bovis* and viral infections, and clinically allows for rapid screening of key targets, which can show a large advantage for medium-sized assays. In the future, experiments will be conducted with viral strains, so that the method can be closer to the clinic and more intuitively reflect the efficiency of the detection of real clinical strains.

## 5 Conclusion

In this study, we have developed a highly efficient, sensitive, and specific qPCR detection method that showed remarkable stability and capability to simultaneously identify six key bovine respiratory pathogens (BRSV, BPIV3, BVDV, BAV3, Mb, and IBRV). In particular, Mb and BAV3 showed the higher detection frequency among all the investigated pathogens. Our findings revealed 56 cases of mixed infections among the positive samples, highlighting the varying prevalence of each pathogen. Based on these test results, drug treatment and immunization programs should be rationalized to cope with the complex pathogen situation of BRDC and to reduce the economic losses caused by BRDC to farmers.

## Data Availability

The original contributions presented in the study are included in the article/Supplementary material, further inquiries can be directed to the corresponding author.

## Supplementary material

Supplementary Figure 1 PCR results of standard plasmids from the six pathogens.

Supplementary Figure 2 Plasmid sequence alignment results of six pathogens.

Supplementary Figure 3 Amplification plots based on the qPCR amplification of six pathogenic plasmids.

Supplementary Figure 4 Amplification plots showing the sensitivity of the qPCR amplification of the six pathogenic plasmids.

Supplementary Figure 5 PCR sensitivity of the six pathogenic plasmids.
