# Supplementary figures and images for: Establishment of a multiplex qPCR assay for the detection of pathogens associated with bovine respiratory disease complex
Source: Front Vet Sci. 2025 Apr 22;12:1594488. doi: 10.3389/fvets.2025.1594488 (PMC12052718; doi:10.3389/fvets.2025.1594488)

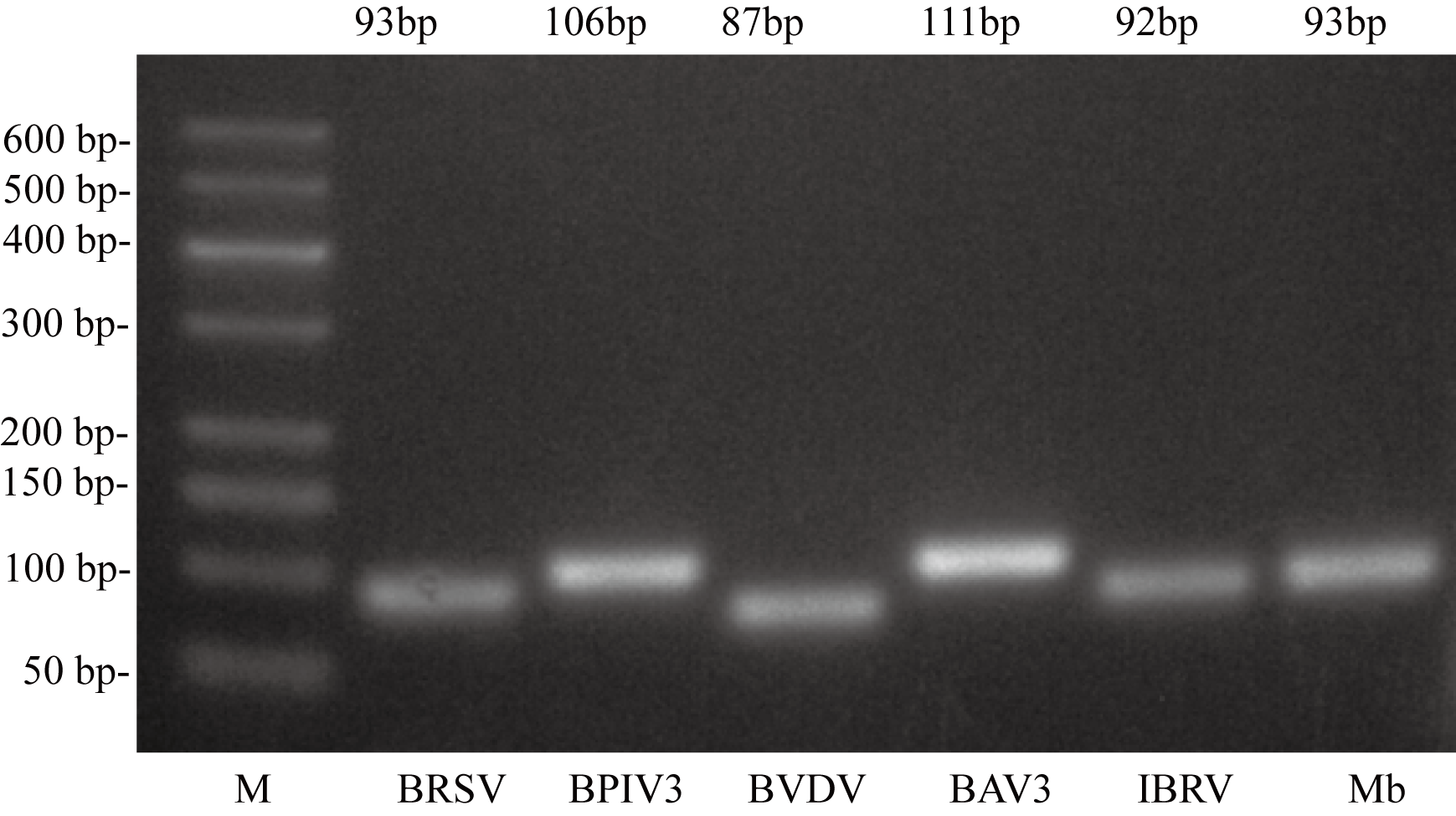

Supplement: Supplementary Figure 1 — PCR results of standard plasmids from the six pathogens. [file Image_1.tif]

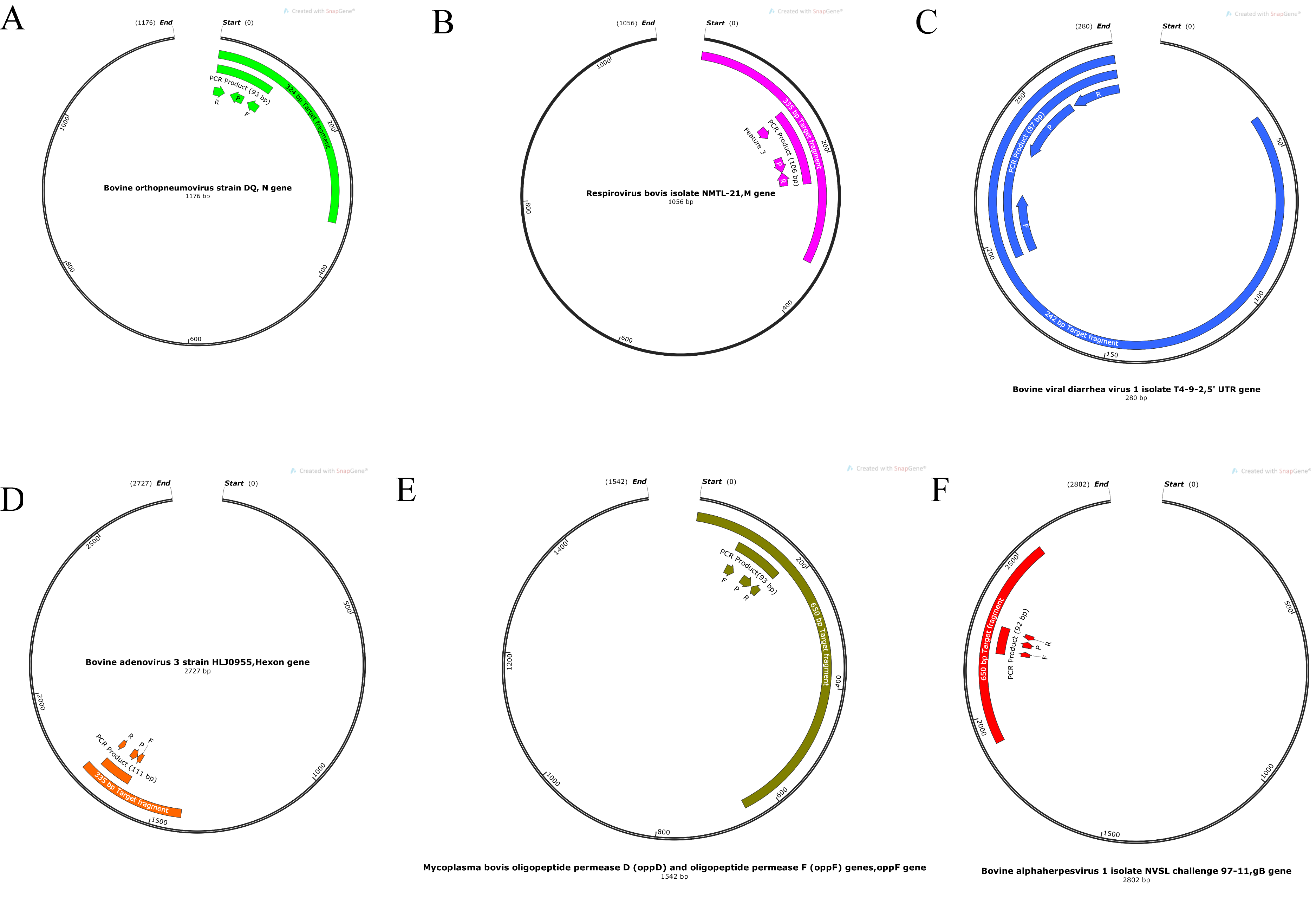

Supplement: Supplementary Figure 2 — Plasmid sequence alignment results of six pathogens. [file Image_2.tif]

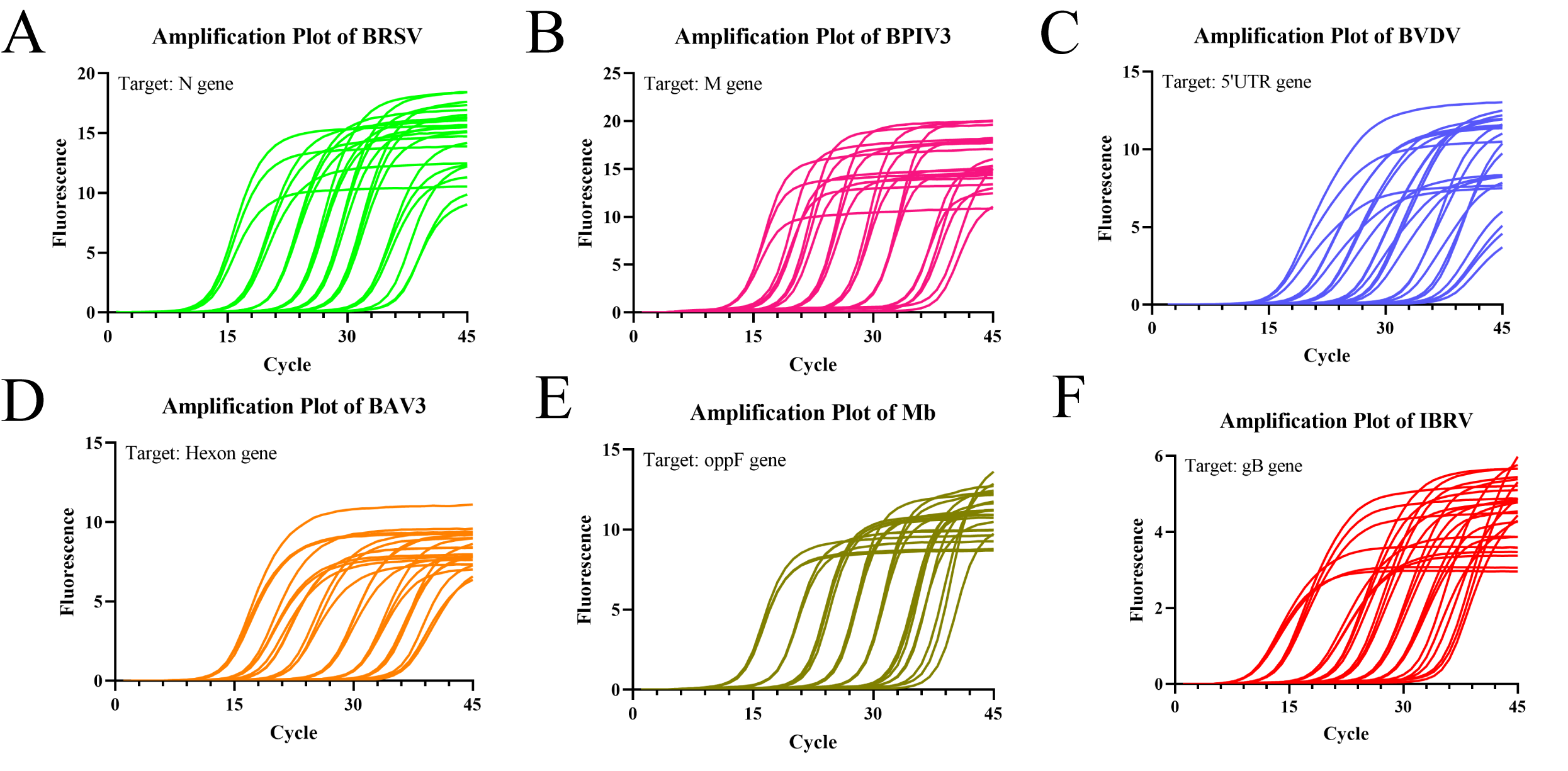

Supplement: Supplementary Figure 3 — Amplification plots based on the qPCR amplification of six pathogenic plasmids. [file Image_3.tif]

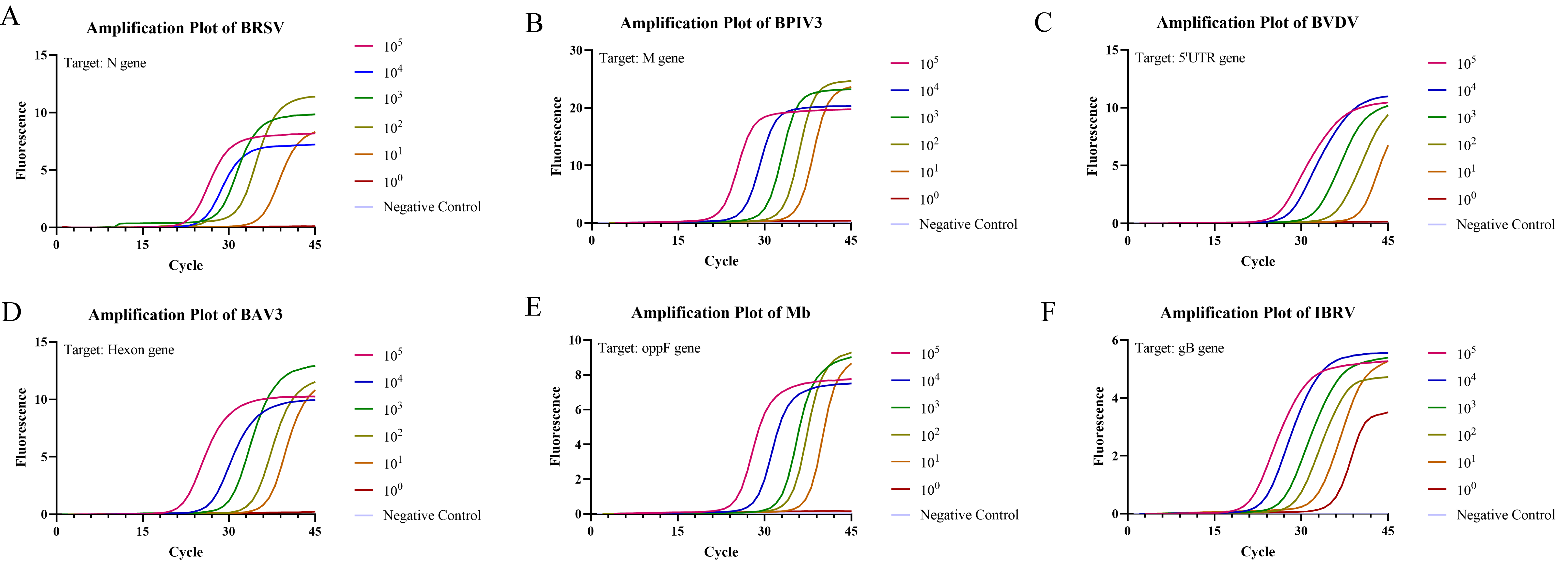

Supplement: Supplementary Figure 4 — Amplification plots showing the sensitivity of the qPCR amplification of the six pathogenic plasmids. [file Image_4.tif]

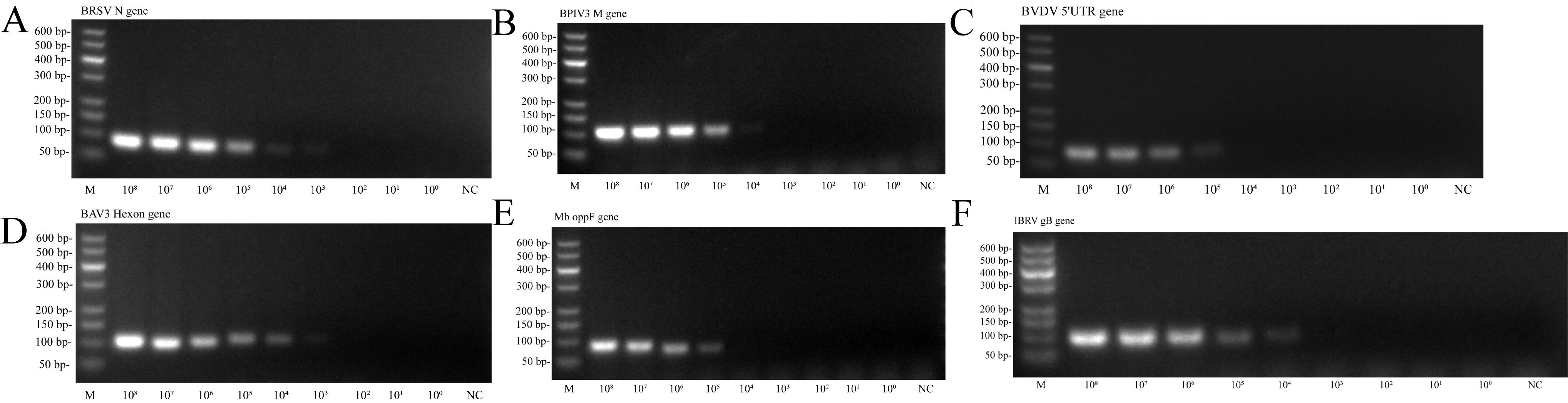

Supplement: Supplementary Figure 5 — PCR sensitivity of the six pathogenic plasmids. [file Image_5.tif]
